# A five years study of antiviral effect of entecavir in Chinese chronic hepatitis B patients

**DOI:** 10.1038/srep28779

**Published:** 2016-07-01

**Authors:** Kehui Liu, Xiaogang Xiang, Rebecca Bao, Rong Chen, Yunye Liu, Jingdong Xie, Qing Guo, Shisan Bao, Qing Xie, Hui Wang

**Affiliations:** 1 Department of Infectious Diseases, Ruijin Hospital, School of Medicine, Shanghai Jiao Tong University, Shanghai, China; 2 Discipline of Anatomy and Histology, School of Medical Sciences and The Bosch Institute, The University of Sydney, Sydney, NSW 2006, Australia; 3 Discipline of Pathology, School of Medical Sciences and The Bosch Institute, The University of Sydney, Sydney, NSW 2006, Australia

## Abstract

Entecavir (ETV) is a potent viral replication inhibitor for chronic hepatitis B (CHB) patients. To investigate the efficacy of ETV in Chinese nucleos(t)ide(NA)-experienced CHB patients. Among 89 CHB patients with ETV monotherapy for ≥6 months, 33/89 (37%) or 56/89 (73%) were NA-naïve or NA-experienced. During a median follow-up of 5.75 years, all NA-naïve CHB patients achieved VR without genotypic ETV-resistance. However, VR was observed in 50/56 (~90%) of NA-experienced CHB patients during a median follow-up of 4.75 years. Antiviral efficacy was not reduced in patients with previous lamivudine (LAM) with/without LAM-resistance (HR 0.465; 95% CI 0.196–1.100; *p* > 0.05) (HR 0.472; 95% CI 0.205–1.091; *p* > 0.05). Patients with a primary treatment failure to adefovir (ADV) had a reduced probability of achieving VR compared to NA-naïve (HR 0.496; 95% CI 0.287–0.857; *p* < 0.01). Previous ADV-experienced patients with a partial VR (HR 1.253; 95% CI 0.429–3.665; *p* > 0.05) did not influence antiviral response to ETV. The antiviral efficacy of ETV is not influenced by previous treatment LAM with/without LAM-resistance. ETV may still be an option in ADV-experienced patients with a partial VR, but not advised in patients with a primary treatment failure to ADV.

The Hepatitis B virus (HBV) remains a major worldwide epidemic, with a high probability of progression to chronic hepatitis, cirrhosis, and hepatocellular carcinoma (HCC). An earlier pivotal study by Chen, *et al*., demonstrated the risk of advancement to cirrhosis, HCC, and liver-related mortality strongly correlates with circulating HBV DNA levels^1^.

Chronic hepatitis B (CHB) patients place a vast drain on the worldwide health industry. Cases of HBV infection have been effectively minimized by the Hepatitis B vaccination, along with two current therapeutic agents approved for the treatment of CHB including nucleos(t)ide analogues (NAs) and interferon α (IFNα)^2^. Substantial improvement in CHB patients has been demonstrated, following the introduction of NAs. In the absence of antiviral drug resistance, continued NAs therapy is able to suppress serum HBV DNA replication and consequently achieve very low or undetectable levels of HBV DNA. The sustained suppression of HBV DNA is associated with delay or even prevention of progression to liver cirrhosis and HCC^3^,^4^.

Entecavir (ETV) is a cyclopentyl guanosine analogue with superior virologic, biochemical, and histological efficacy compared with other NAs^5^,^6^,^7^. It is widely used as a first-choice NA for CHB patients, due to the low rate of genotypic resistance in NA-naïve CHB patients through five years of continuous ETV monotherapy^8^,^9^. ETV is less potent and the rate of genotypic resistance is increased in lamivudine (LAM)-refractory CHB patients^10^,^11^, which is supported by clinical treatment data from European multicenter studies^12^,^13^.

Chinese clinicians face large challenges due to the increasing number of CHB patients, particularly within the Han race. These CHB patients have been experiencing treatment failures through different NA-therapeutic regimens, due to inadequate response, non-compliance, or financial barriers. The long-term outcomes of nucleoside-experienced Chinese patients treated with ETVs for more than five years are still unclear. The aim of this study was to assess the efficacy of ETV in CHB patients, focusing on NA-experienced groups.

## Results

The baseline characteristics of the 89 patients were summarized (Table 1). Overall, the median follow-up was 63 (12–75) months. Sixty-seven (75%) patients were hepatitis B envelop antigen^+^ (HBeAg^+^), mean HBV DNA of all patients was 5.9 ± 1.8 log_10_ IU/mL, and mean alanine aminotransferase (ALT) was 80 ± 136.2 IU/L. NA-experienced patients with HBeAg^+^ were significantly more than NA-naïve patients with HBeAg^+^ (*p* < 0.01).

### Efficacy of ETV in NA-naïve CHB patients

**Figure 1:**
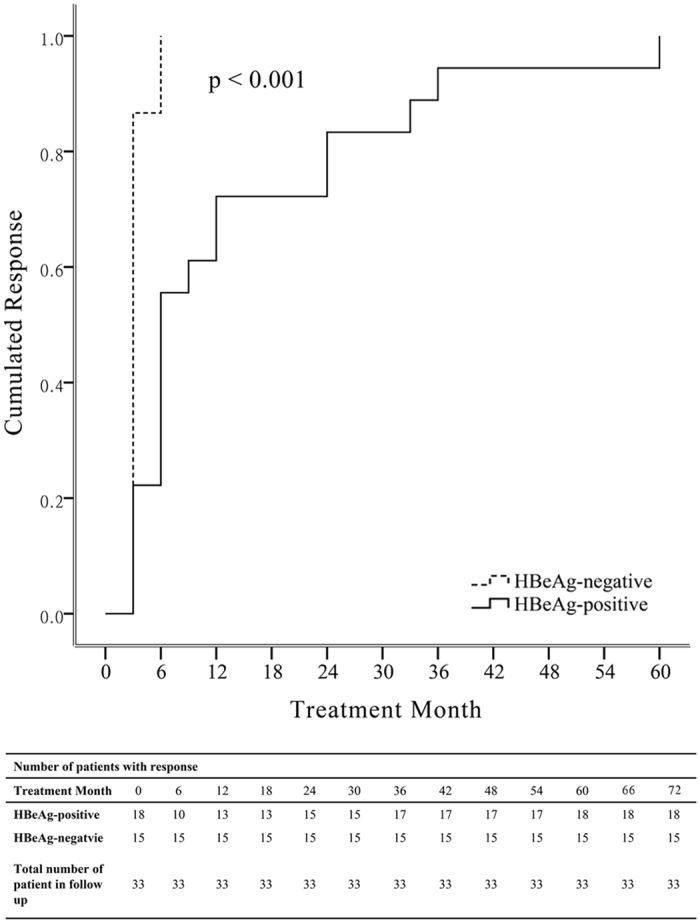
Kaplan-Meier curve for the probability of response for 33 NA-naïve patients according to HBeAg status at baseline. *P* value was determined using log-rank testing.

Overall, 33/89 (37%) NA-naïve patients all achieved virologic response (VR) after a median follow-up of 69 (60–75) months (Table 2). The cumulative probability of achieving VR in HBeAg^+^ patients (n = 18), at 3, 6 and 12 months were 22%, 56% and 72%, respectively (Fig. 1). On the other hand, the cumulative probability of VR response in hepatitis B envelop antigen^−^ (HBeAg^−^) patients (n = 15) at 3, 6 and 12 months were 87%, 100%, and 100%, respectively (Fig. 1). Six of 18 (33%) HBeAg^+^ patients lost HBeAg following a median treatment duration of 72 (63–75) months, and 5/18 (28%) patients seroconverted to hepatitis B envelop antibody (anti-HBe). No hepatitis B surface antigen (HBsAg) loss and virologic breakthrough were detected in NA-naïve patients during follow-up (Table 2). There was no significantly different anti-viral outcome between cirrhotic (n = 5) and chornic hepatitis (n = 23) in NA-naïve patients.

### Efficacy of ETV in NA-Experienced CHB patients

There was 56/89 (63%) patients with NA-experience in the current study. VR was developed in 50/56 (~90%) of NA-experienced patients during a median follow-up of 57 (12–75) months. The cumulative probability of achieving VR in NA-experienced patients, at 1, 2, 3, 4 and 5 years were 61%, 64%, 73%, 88% and 88%, respectively. Thirty-nine (~70%) subjects were directly switched to ETV monotherapy after failure to preceding NA therapy. The median duration between the end of previous NA treatment and the start of ETV monotherapy was 9 (1–74) months for the rest of 17 patients. No significantly different anti-viral outcomes were detected between cirrhotic (n = 8) and chronic hepatitis (n = 37) in NA-experienced patients based on liver biopsy, however another 11 NA-experienced patients lacked liver fibrosis data.

**Figure 2:**
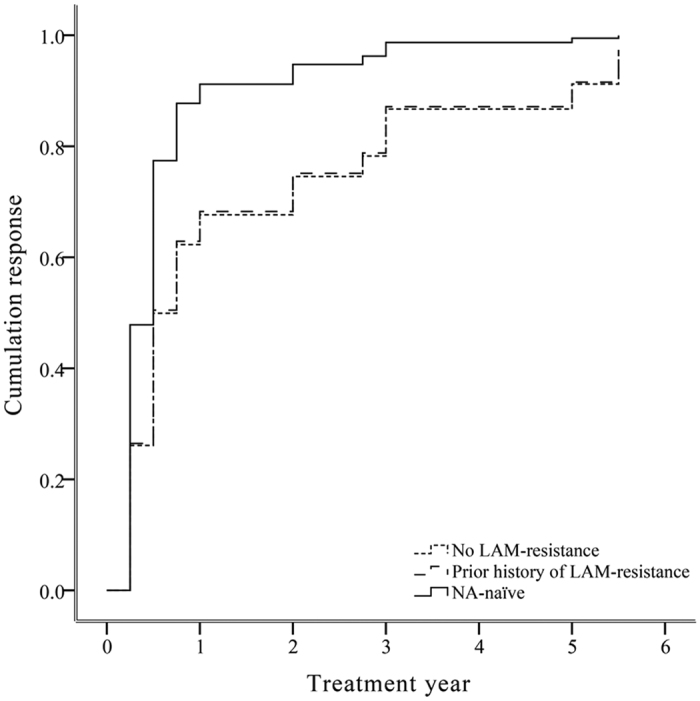
Adjusted estimated survival curve for the cumulative probability of achieving virologic response for NA-naïve and different subsets of LAM-experienced CHB patients. Based on the Cox’s model for baseline HBV DNA, ALT level and HBeAg status.

Twenty-one (21/56, 38%) patients had previous LAM treatment, and median time between the end of LAM treatment and the start of ETV treatment was 6 (0–19) months. The cumulative probability of achieving VR in LAM-experienced patients, at 1, 2, 3, 4 and 5 years were 62%, 62%, 67%, 67% and 67%, respectively. Ten (10/21, 48%) patients developed LAM-resistance, of which 3/10 (33%) subjects displayed LAM-resistance even at the beginning of ETV monotherapy. The efficacy of ETV in different subgroups of LAM-experienced patients was presented (Table 2). Antiviral efficacy of ETV was not decreased by previous LAM treatment after adjusted for baseline viral load, HBeAg status and ALT level. This was supported by the findings that there was no significantly different antiviral efficacy of ETV between NA-naïve and NA-experienced patients with or without presence of LAM-resistance (HR 0.465; 95% CI 0.196–1.100; *p* > 0.05) (HR 0.472; 95% CI 0.205–1.091; *p* > 0.05) (Fig. 2).

**Figure 3:**
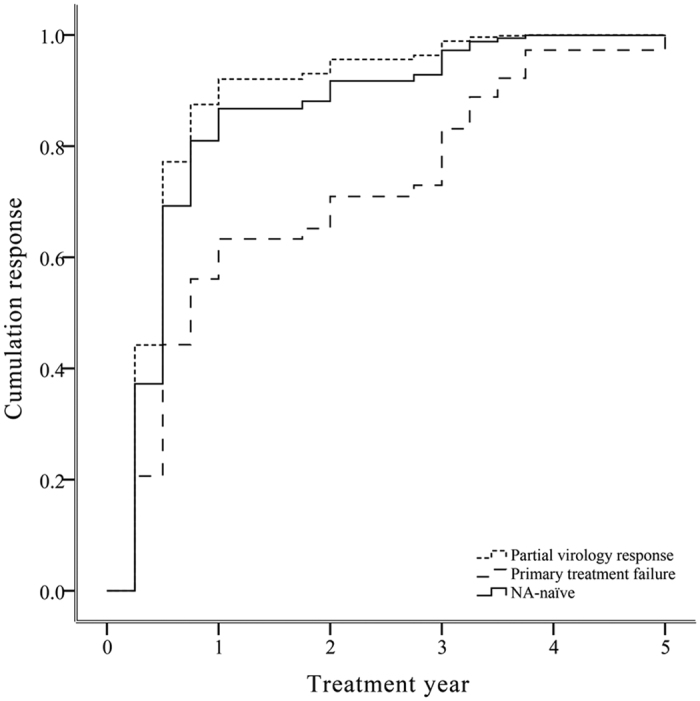
Adjusted estimated survival curve for the cumulative probability of achieving virologic response for NA-naïve and different subsets of ADV-experienced CHB patients. Based on the Cox’s model for baseline HBV DNA, ALT level and HBeAg status.

Forty-three (43/56, 77%) patients with previous adefovir (ADV) treatment (Table 1), of whom 8 patients initially received LAM monotherapy, and 2 patients stopped antiviral therapy after 2 or 12 months of ADV monotherapy, respectively. The cumulative probability of achieving VR in ADV-experienced patients, at 1, 2, 3, 4 and 5 years were 58%, 63%, 74%, 93% and 93%, respectively. Rescue therapy of ETV was evaluated in the ADV-treated patients who did not receive LAM and directly switched to ETV (Table 2). After adjusted baseline viral load, HBeAg status and ALT level, there was no significant influence of previous ADV therapy with a partial virology response on antiviral response to ETV when compared to NA-naïve subjects (HR 1.253; 95% CI 0.429–3.665; *p* > 0.05) (Fig. 3). Furthermore, ADV primary treatment failure patients reduced the probability of achieving VR compared to NA-naïve patients (HR 0.496; 95% CI 0.287–0.857; *p* < 0.01) (Fig. 3).

### Resistance

During a median follow-up of 63 (12–75) months, 10 of 89 (11%) patients experienced a virologic breakthrough, and 9 of 10 (90%) experienced ETV-resistance. Among 56 NA-experienced patients 50 patients achieved VR during the follow-up. In these 6 non VR patients, the serum HBV DNA levels increased more than 1 log10 IU/ml compared to the nadir (lowest value) HBV DNA level on therapy at least two occasions. Thus, the 6 non VR patients were classified as virological breakthrough, based on the guideline of EASL. Additionally, four out of 50 VR patients experienced a virological breakthrough at diffierent times, resulting in a total patient count of 10/56 with virological breakthrough. Four out of nine ETV-resistance patients switched to ETV plus ADV regimen, while other five patients remained ETV monotherapy, due to financial difficulty. Three of these ten virologic breakthrough patients had a prior history of developed LAM-resistance. Among these three patients, two had LAM-resistant mutation (rtL180M) at the start of ETV monotherapy, and further developed ETV-resistant mutations (rtT184A, rtM204V) during the follow-up. One patient with mutation (rtM204I) related to both LAM-resistance and ETV-resistance at baseline, achieved virologic response at 36 weeks and experienced a virologic breakthrough at 96 weeks. Unfortunately, this patient changed to ETV plus ADV regimen at very late stage (204 weeks) due to financial difficulty and developed HCC at 216 weeks. Only 5/9 ETV resistant patients achieved VR. None of NA-naïve patients experienced a virologic breakthrough during follow-up compared with NA-experienced patients.

### Safety

No severe renal adverse event was observed in these patients over the period of the current study. The renal function was evaluated with blood urea and creatinine levels in all patients. There were only 11 patients with transient mild elevations of blood urea and creatinine levels, due to over exercises (such as climbing, and running) and/or labour related work. Renal function was observed to return to normal levels after ceasing excessive exercises. None of the patients developed clinically evident elevated blood lactate or creatine kinase.

### HBsAg decline

**Figure 4:**
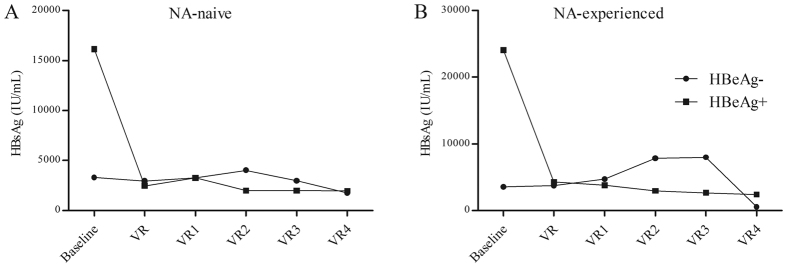
HBsAg Decline of achieving virologic response for NA-naïve and NA-experienced CHB patients. (**A**) HBsAg decline of achieving virologic response for NA-naïve CHB patients, (**B**) HBsAg decline of achieving virologic response for NA-experienced CHB patients. (VR1: one year after achieving VR, VR2: two year after achieving VR, VR3: three year after achieving VR, VR4: four year after achieving VR).

In total, 83/89 (94%) patients achieved VR during a median follow-up of 57 (12–75) months. HBsAg level was selected at baseline in all HBV patients, and were taken place routinely at every visit. In HBeAg^+^ patients, the mean HBsAg decline was 16124 to 2456 IU/mL (Baseline to VR) for NA-naïve patients and 24037 to 4264 IU/mL for NA-experienced ones. While in HBeAg^-^ patients, the mean HBsAg decline was 3239 to 2945 IU/mL and 3544 to 3719 IU/mL for NA-naïve and NA-experienced ones, respectively (Fig. 4).

## Discussion

We have demonstrated for the first time, the efficacy of long-term ETV treatment in Chinese NA-experienced CHB patients. Previous treatment of LAM with/without LAM-resistance does not influence the efficacy of ETV treatment. Furthermore, ETV may still be an option in ADV-experienced patients with a partial VR, but is not advised in patients with a primary treatment failure to ADV therapy. LAM and ADV are still wildly used for treatment of CHB in the developing countries. Our current findings on LAM and ADV in relation to ETV treatment may provide guideline in clinical intervention.

ETV has probably superior virologic, biochemical, and histological efficacy compared to other current NAs agents, arguably equal efficacy with tenofovir disoproxil fumarate (TDF)^14^. The five-year ETV clinical trial (ETV-022) demonstrated that 94% had HBV DNA < 300 copies/mL, 80% had normal ALT levels, and 23% achieved HBeAg seroconversion in NA-naïve CHB patients in Europe^15^. In addition, >90% ETV treated NA-naïve Chinese CHB patients achieved HBV DNA undetectable after 2 years, VP increased at 12 and 24 weeks, and HBeAg seroconversion achieved 15.4% at year three^9^. The above findings^9^,^15^,^16^ support our current study, which demonstrated that all NA-naïve patients achieved VR at year five without genotypic ETV-resistance. HBV DNA was reduced substantially accompanied by increased VR at 12 and 24 weeks, in addition to increased rate of ALT normalization. Moreover, our data also showed that the rate of HBeAg seroconversion in the Chinese patients was 17%, 28% or 33% at year two, three or five, respectively, which are similar with the results of previous studies^17^. The combined data from our study and previous studies demonstrate that ETV is highly effective in NA-naïve patients. It has been reported that ETV has rare occurrence of resistance in NA-naïve patients^9^, which is consistent with our findings over a five-year period. Thus detecting mutations for pre-existing resistance to ETV in NA-naïve patients is of limited significance from financial and practical point of view.

LAM has been routinely used as a first-line therapy for CHB patients, however has incurred major limitations due to growing resistance over the past decade^18^. Resistance is usually associated with a rebound in viral load and often associated with exacerbation of hepatitis^19^. Increasing number of treatment failure to different NA-treatment regimens poses a growing problem in daily clinical practice.

The effect of ETV monotherapy in CHB patients has been categorized into two groups: NA-naïve and NA-experienced in Reijnders’s study^12^. The presence of LAM-resistant mutations at the start of ETV was significantly associated with a reduced probability of achieving VR compared to LAM-naïve patients. Previous LAM treatment without development of LAM-resistance, or with a prior history of LAM-resistance, did not influence the antiviral response over a one-year period^12^. This data is consistent with our five-year study, demonstrating antiviral efficacy was not decreased by previous LAM treatment, with or without LAM-resistance. It was reported that ETV resistance developed more frequently in LAM-treated CHB patient over an 18-month period, but no prior history of LAM-resistance did not affect the development of ETV resistance^20^. Our data showed that among nine developed ETV resistance patients, three did not have LAM-resistance, but four had a prior history of LAM-resistance. Of those four patients, three subjects expressed detectable LAM-resistant mutations at the initiation of ETV monotherapy. Our five-year study suggested that LAM-resistant CHB patients with ETV treatment results in a high probability of progression to ETV-resistance, particularly in the group with previous detectable LAM-resistant mutation at the start of ETV therapy.

ADV, an established medication for the treatment of CHB, has been widely used in China in the past decade. ADV has additionally been associated with a high rate of primary treatment failure, defined as less than a 2-log reduction in viral load after six months of therapy, and a high rate of antiviral resistance^2^,^21^,^22^. The efficacy of ETV in CHB patients previously treated with ADV has been relatively un-studied, particularly in cases with primary treatment failure^23^,^24^. It has been also reported that the effects of ETV monotherapy in previous ADV-treated CHB patients demonstrating that partial responders do not display as good effect as ADV-complete responders^25^,^26^. Interestingly, previous treatment with ADV and presence of ADV-resistant mutations does not influence the potency of ETV^12^. Our five-year-period study established that there was almost equal effect with ETV treatment between the CHB patients prior to ADV therapy with a partial virology response and NA-naïve patients. Such discrepancies may be due to a difference in time period (three *vs* five years) and also in different populations (Caucasians *vs* Chinese). More importantly, we found that CHB patients with a primary treatment failure history had a reduced probability of achieving VR compared to NA-naïve patients. Therefore, the response to prior treatment is necessary for ADV-experienced patients before starting ETV monotherapy.

In our current study, all ADV-experienced and NA-naïve patients were treated with 0.5 mg ETV; whereas all LAM-experienced patients were treated with 1 mg ETV monotherapy, while NA-naïve patients were treated with 0.5 mg ETV. This data suggests that 1 mg of ETV provides no more obvious anti-viral benefit in LAM-experienced patients compared to that of NA-naïve patients with 0.5 mg ETV treatment. During the follow up, there was no significantly different follow-up time between LAM-experienced and naïve groups, however a significant difference was detected between ADV-experienced and naïve groups. As those non-VR patients had already experienced viral breakthrough within three years, the extended follow-up period may not contribute to the major cause of reduced VR among NA-experienced patients. In this study, no significant difference was observed between cirrhotic and chronic hepatitis patients, however this is more likely due to a relatively small number of patients. A large cohort study is currently being investigated.

Limitations of our study include the heterogeneous group and relative small sample size. Cox regression has been applied to correct for confounders as treatment duration, HBV DNA, HBeAg status. Nevertheless, the Out-patient Department of Infectious Diseases in Shanghai Ruijin Hospital with consultation number is 12000 per month, one of the biggest in the China, and covers large areas of patients. Thus, the patients still represent of clinical practice and make it possible to compare different groups of NA-experienced patients. We appreciate the small scale of the study, despite spanning a five-year period. A large scale study with an expanded population size, and extended period of time is currently in the process of being investigated.

Despite the influence of previous IFN exposure on the efficacy of ETV, only 17 (19%) of patients had prior experience of IFN treatment within our current study. The impact of IFN to long-term ETV therapy is currently lacking in understanding, and still requires further exploration.

In conclusion, ETV proved to be efficacious in NA-naïve patients. ETV may still be an option in ADV-experienced patients with a partial virology response, however it is not recommended in patients with a primary treatment failure to ADV therapy.

## Methods

### Study population

Adult CHB patients (n = 89) with consecutive HBsAg^+^ for at least six months, were recruited for this cohort study. Patients were consecutively treated with ETV monotherapy between March 2007 and May 2013, in the Outpatient, Department of Infectious Diseases, Shanghai Ruijin Hospital. The exclusion criteria included: 1) Co-infected with human immunodeficiency virus (HIV), hepatitis C virus (HCV) or hepatitis D virus (HDV); 2) Undergone liver transplantation before the start of ETV treatment; 3) HCC, autoimmune liver disease or alcoholic fatty liver disease.

This study complies with the declaration of Helsinki, and the study protocol was approved by the Ethics Committee of Ruijin Hospital. Written informed consent was obtained from all patients according to standards of the local ethics committees.

### Follow-up participants

All subjects were monitored at the discretion of the treating physician at least every three months. Routine biochemistry (ALT, bilirubin, albumin, serum creatinine) and virologic tests (HBV DNA level, HBsAg, hepatitis B surface antibody (anti-HBs), HBeAg, anti-HBe) were performed at each visit. Genotypic analysis was determined at baseline in all NA-experienced HBV patients, and in case of virologic breakthrough, defined as a confirmed increase in HBV DNA level of more than 1 log10 IU/ml compared to the nadir (lowest value) HBV DNA level on therapy at least two occasions^27^. Genotypic analysis was performed at baseline in NA-naïve subjects only in the patients with ETV-resistant mutations during follow-up. HBV genotype was determined at baseline. The severity of liver fibrosis was confirmed with biopsy or B ultrasound.

### Endpoints

The primary outcome was VR, defined as serum HBV DNA levels <100 IU/ml during the on-treatment follow-up period. Secondary endpoints were HBeAg loss and seroconversion for HBeAg-positive patients, HBsAg loss and seroconversion, ALT normalization in patients with abnormal ALT at baseline, or emergence of ETV-related mutations.

### Laboratory tests

ALT, bilirubin, albumin and prothrombin time *et al*. were measured routinely at the Department of Biochemistry in Ruijin Hospital. HBsAg, anti-HBs, HBeAg and anti-HBe were determined using commercial ELISA kits (Abbott Diagnostics, IL). Serum HBV DNA levels were measured using qPCR, Roche Amplicor (Roche Diagnostic Systerms, Branchburg, NJ, USA). A conversion factor of 5.26 was used for conversion of copies/ml to IU/ml. Presence of HBV polymerase gene mutations was determined by direct sequencing. HBV genotypes were assessed by direct sequence alignment of the overlapping hepatitis B surface antigen with HBV sequenced derived from GenBank.

### Statistical analysis

Continuous variables were expressed as means ± standard deviation or median (interquartile range) where appropriate. Follow-up times were calculated from the date of ETV treatment initiation to the date of event. Cumulative probabilities of different endpoints were estimated by Kaplan-Meier ananlysis. Cox regression model was used to analyze which of the following baseline factors were associated with VR to ETV monotherapy: prior treatment with LAM, prior history of LAM-resistance, prior history of partial virology response to ADV, and prior history of primary treatment failure to ADV. All statistical tests are two-side, and *p*-value < 0.05 was considered to be statistically significant. SPSS version 22.0 was used for all statistical analysis (SPSS Inc., Chicago, IL, USA).

## Additional Information

**How to cite this article**: Liu, K. *et al*. A five years study of antiviral effect of entecavir in Chinese chronic hepatitis B patients. *Sci. Rep.* **6**, 28779; doi: 10.1038/srep28779 (2016).

## Figures and Tables

**Table 1:** Baseline characteristics of the study population.

	ALL patients n = 89	NA-naïve n = 33	NA-experienced n = 56	*p*-Value
Age(year)	44 ± 11	49 ± 9	42 ± 11	<0.01
Gender (male%)	70 (79%)	22 (68%)	48 (86%)	<0.05
BMI (kg/m^2^)	23 ± 3.1	23.4 ± 2.9	23 ± 3.2	n.s.
ALT (IU/L)	80 ± 136.2	82 ± 51.1	79 ± 166.1	n.s.
AST (IU/L)	63 ± 110.2	63 ± 33.6	63 ± 135.5	n.s.
TBIL (μmol/L)	20 ± 12.8	17 ± 5.8	21 ± 15.3	n.s.
ALB (g/L)	44 ± 3.2	42 ± 2.6	44 ± 3.3	<0.01
HBsAg (IU/mL)	13374 ± 23703	8393 ± 13465	15692 ± 27019	n.s.
HBeAg-positive	67 (75%)	18 (55%)	48 (86%)	<0.01
HBV-DNA (log_10_IU/ml)	5.9 ± 1.8	6.2 ± 1.9	5.7 ± 1.7	n.s.
Cirrhosis/chronic hepatitis	13/65	5/28	8/37	n.s.
Genotype (n = 76)				
B	19 (25%)	5 (19%)	14 (28%)	n.s.
C	52 (68%)	19 (73%)	33 (66%)	
Other	5 (7%)	1 (8%)	3 (6%)	
Previous treatment with (peg) IFN	17 (19%)	5 (15%)	12 (21%)	n.s.
Previous LAM treatment				
LAM-experienced	21 (24%)		21 (38%)	
Prior history of LAM-resistance	10 (11%)		10 (18%)	
Previous ADV treatment				
ADV-experienced	43 (48%)		43 (77%)	
Partial virology response	8 (9%)		8 (14%)	
Primary treatment failure	32 (3%)		32 (57%)	
Previous treatment with LdT	2 (2%)		2 (4%)	
Follow up (month)		69 (60–75)	57 (12–75)	<0.0001
LAM-experienced			69 (60–75)	n.s.
ADV-experienced			51 (12–72)	<0.0001

(ALT: alanine aminotransferase; AST: aspartate aminotransferase; TBIL: total bilirubin; ALB: albumin; ADV: adefovir dipivoxil; IFN: interferon; LdT: Telbivudine).

**Table 2:** Virologic and biochemical response to entecavir.

	NA-naïve (n = 33)	LAM-experienced (n = 21)		ADV-experienced (n = 33)	
		No LAM-resistance (n = 11)	Prior history LAM-Resistance (n = 10)	Partial virology response (n = 4)	primary treatment failure (n = 29)
Baseline HBV DNA(log_10_IU/ml)	6.2 ± 1.9	7.0 ± 1.5	5.4 ± 1.9	3.5 ± 1.4	5.6 ± 1.4
Median follow-up (y)	5.75 (5–6.25)	6 (1.5–6.25)	5 (1.75–6)	5.75 (5–6)	4.25(1–5.75)
ALT normalization	18/18 (100%)	2/3 (67%)	3/3 (100%)	0/0	6/8 (75%)
Virologic response					
1 year	28/33 (85%)	7/11 (64%)	6/10 (60%)	4/4 (100%)	14/29 (48%)
2 year	30/33 (91%)	7/10 (70%)	6/8 (75%)	4/4 (100%)	16/28 (57%)
3 year	32/33 (97%)	7/10 (70%)	6/7 (86%)	4/4 (100%)	20/28 (71%)
4 year	32/33 (97%)	8/10 (80%)	7/7 (100%)	3/3 (100%)	27/27 (100%)
5 year	33/33 (100%)	8/10 (80%)	6/6 (100%)	3/3 (100%)	10/10 (100%)
Virologic breakthrough					
1 year	0/33 (0%)	0/11 (0%)	0/10 (0%)	0/4 (0%)	0/29 (0%)
2 year	0/33 (0%)	2/10 (20%)	2/8 (25%)	0/4 (0%)	0/29 (0%)
3 year	0/33 (0%)	3/10 (30%)	3/7 (43%)	0/4 (0%)	0/29 (0%)
4 year	0/33 (0%)	3/10 (30%)	3/7 (43%)	1/4 (25%)	0/29 (0%)
5 year	0/33 (0%)	3/10 (30%)	4/6 (67%)	1/4 (25%)	0/29 (0%)
6 year	0/33 (0%)	3/10 (30%)	4/6 (67%)	1/4 (25%)	1/29 (3%)
Genotypic ETV-resistance	0/33 (0%)	3/11 (27%)	4/10 (40%)	1/4 (25%)	1/29 (3%)
HBeAg loss	6/18 (33%)	1/8 (13%)	1/7 (14%)	1/4 (25%)	6/27 (22%)
1 year	1/18 (6%)	0/8 (0%)	1/7 (14%)	0/4 (0%)	1/26 (4%)
2 year	3/18 (17%)	1/7 (14%)	1/7 (14%)	0/4 (0%)	2/25 (8%)
3 year	5/18 (28%)	1/7 (14%)	1/7 (14%)	0/4 (0%)	4/25 (16%)
4 year	5/18 (28%)	1/7 (14%)	1/7 (14%)	0/4 (0%)	4/24 (17%)
5 year	6/18 (33%)	1/7 (14%)	1/7 (14%)	1/4 (25%)	3/10 (30%)
HBsAg loss	0/33 (0%)	0/11 (0%)	0/10 (0%)	0/4 (0%)	0/29 (0%)

To explore the role of ETV as rescue therapy for ADV-treated patients, the antiviral effect of ETV is described for those patients, who did not receive LAM therapy and were directly switched to ETV monotherapy (n = 33/43, 77%).
